# Evaluation of the Prognostic Capacity of a Novel Survival Marker in Patients with Sinonasal Squamous Cell Carcinoma

**DOI:** 10.3390/nu14204337

**Published:** 2022-10-17

**Authors:** Faris F. Brkic, Stefan Stoiber, Sega Al-Gboore, Clemens Quint, Julia Schnoell, Alexandra Scheiflinger, Gregor Heiduschka, Markus Brunner, Lorenz Kadletz-Wanke

**Affiliations:** 1 Department of Otorhinolaryngology and Head and Neck Surgery, Medical University of Vienna, 1090 Vienna, Austria; 2 Department of Pathology, Medical University of Vienna, 1090 Vienna, Austria; 3 Christian Doppler Laboratory for Applied Metabolomics, Medical University of Vienna, 1090 Vienna, Austria

**Keywords:** survival index, sinonasal squamous cell carcinoma, prognostic marker, survival, outcome

## Abstract

Sinonasal squamous cell carcinoma (SNSCC) is a malignant tumor associated with poor survival, and easily obtainable prognostic markers are of high interest. Therefore, we aimed to assess the prognostic value of a novel survival index (SI) combining prognostic values of clinical (T and *N* classifications and invasion across Ohngren’s line), inflammatory (neutrophil-to-lymphocyte ratio), and nutritional (albumin and body-mass index) markers. All patients with primarily treated SNSCC between 2002 and 2020 (*n* = 51) were included. Each of the six SI components was stratified into a low- (0) and high-risk (1) categories. Subsequently, the cohort was stratified into low- (SI of 0–2) and high-risk SI groups (SI of 3–6). Overall survival (OS) and disease-free survival (DFS) were compared between patients with low- and high-risk SI. The log-rank test was used to test for statistical significance. Overall, the mortality rate was 41.2% (*n* = 21), and the recurrence rate was 43.1% (*n* = 22). We observed significantly better OS in patients with low-risk SI (*n* = 24/51, 47.1%, mean OS: 7.9 years, 95% confidence interval (CI): 6.3–9.6 years) than in high-risk SI (*n* = 27/51, 52.9%, mean OS: 3.4 years, 95% CI: 2.2–4.5 years; *p* = 0.013). Moreover, we also showed that patients with low-risk SI had a longer DFS than patients with high-risk SI (mean DFS: 6.4, 95% CI: 4.8–8.0 vs. mean DFS: 2.4 years, 95% CI 1.3–3.5, *p* = 0.012). The SI combines the prognostic capacity of well-established clinical, radiologic, inflammatory, and nutritional prognosticators and showed prognostic potential in our cohort of SNSCC patients.

## 1. Introduction

Malignant tumors of nasal and paranasal sinuses are rare and comprise up to 5% of all head and neck malignancies [1]. The most common histologic subtype is sinonasal squamous cell carcinoma (SNSCC), which makes up more than half of all nasal and paranasal malignancies. Other histologic variants are adenocarcinoma, adenoid-cystic carcinoma, melanoma, and sinonasal undifferentiated carcinoma [2]. Patients with SNSCC generally have poor survival outcomes, which have not significantly improved in recent years [3]. The reported 5-year overall survival (OS) ranges from 30% to 50% [1,3,4]. Predicting patient outcome is still hard for this disease due to the lack of established prognostic markers. Moreover, most prognosticators that are available for SNSCC were tested only on a limited patient population due to the infrequent occurrence pattern of SNSCC. Research on prognostic markers therefore seems particularly essential, as it may help with stratifying high-risk patients who would benefit from more aggressive therapy regimens and more frequent clinical follow-ups.

Recently, the prognostic relevance of nutritional and cachexia markers in cancer patients has attracted considerable attention. Two well-established markers are the serum albumin and body-mass index (BMI). These were combined (together with neutrophil-to-lymphocyte ratio (NLR)) in the advanced lung cancer inflammation index (ALI) by Jafri et al. [5]. The predictive capacity of this prognosticator has been shown for non-small-cell lung cancer [5], colorectal cancer [6], lung adenocarcinoma [7], and pancreatic cancer [8]. Moreover, several studies have reported its prognostic relevance in head and neck malignancies [9,10,11,12]. There are several hypotheses regarding the association of hypoalbuminemia with worse outcomes in cancer. First, Fearon et al. proposed an increase in albumin degradation in cancer patients and rejected the idea of a lower synthesis of albumin as an underlying mechanism [13]. Furthermore, it was hypothesized that the lowered albumin level in tumor patients is caused by weight loss, particularly cellular weight loss [14]. On the other hand, a higher BMI can potentially indicate higher nutritional reserves that could certainly promote endurance during cancer treatment. This is particularly relevant in head and neck cancer patients, who often have dysphagia due to tumor localization and other eating-related problems, such as radiation-induced mucositis, oral pain, chemotherapy-induced appetite loss, etc. [15]

On the other hand, the association of inflammation within the tumor microenvironment and tumorigenesis is already well-established [16]. Therefore, many studies have analyzed the prognostic relevance of various inflammatory and hematologic markers in several tumor entities. In particular, a high pretreatment NLR and the platelet-to-lymphocyte ratio predicted worse survival outcomes in patients with sinonasal cancer. Particularly for SNSCC, high NLR was associated with impaired OS and disease-free survival (DFS) [17]. Specifically, the NLR should reflect the degree of the cancer-associated inflammation. The research on this topic started when the role of leukocytes in cancer development had only recently been proposed. The discovery of leukocytes in the tumor environment in the nineteenth century led to subsequent research. Nowadays, the role of inflammation in cancer promotion and development is well-known, and inflammation is recognized as one of the hallmarks of cancer [18]. In a previous study, our study group analyzed the prognostic value of NLR, BMI, and ALI for SNSCC, and was able to show that only low BMI independently predicted worse survival [19]. However, a limited number of patients were included in the study, which might have contributed to missing some significant results.

In addition to the T and *N* classification, as typical clinical prognosticators reflecting tumor size and nodal involvement [20], tumor invasion across Ohngren’s line (OL) seems to have a negative impact on survival [21]. The OL divides the maxillary sinus and passes from the medial border of the orbita to the mandibular arch [20]. Combining the values of noted clinical prognostic markers could potentially synergistically increase the overall prognosticator capacity, in particular for SNSCC.

Generally, the literature remains sparse in regards to easily obtainable prognostic markers for patients with SNSCC. This and the fact that mortality and recurrence rates in this disease remain high underline the need for new outcome prognosticators, which could facilitate filtering-out of high-risk patients. The current literature provides evidence of combining the prognostic values of different markers into one prognostic score. For example, Hum et al. proposed a “PRO-MAC” prognostic model for cancer in general, combining several clinical prognostic markers [22]. Furthermore, we identified several studies with these efforts for specific cancer types as well. First, Repo et al. provided evidence of a solid prognosticator combining several clinical prognostic markers in breast cancer [23]. Similar efforts for colorectal cancer could be identified, as reported by Mahar et al. [24]. According to these findings and efforts for cancer in general, as well as specific cancer entities, we concluded that such composite scores combining the prognostic values of already established clinical, nutritional, and hematologic markers should certainly be investigated in SNSCC. As previously noted, the survival rates of SNSCC have remained poor over the last decades without any relevant improvements. Therefore, these patients would undoubtedly benefit from a pretherapeutic risk stratification, which would enable filtering-out of high-risk patients. This patient group could then be evaluated for more aggressive treatment options and more frequent clinical post-therapeutic follow-up regimes. A prognostic marker that is feasible and easily obtainable (through routinely conducted imaging and blood sampling) is surely warranted and helpful.

Based on the previous findings and apparent prognostic relevance of noted clinical and inflammatory markers in SNSCC and cancer patients in general, we aimed to combine their prognostic capacity into one single score. Therefore, we propose the novel survival index (SI), an outcome marker combining the prognostic values of hematologic inflammation and cachexia markers (NLR, BMI, and albumin) with clinical characteristics (T classification, *N* classification, and tumor invasion of the OL) and aimed to test its prognostic significance in patients with SNSCC.

## 2. Materials and Methods

In this retrospective chart study, all patients with a histologically verified SNSCC diagnosed and primarily treated at our center between 1st January 2002 and June 2020 were included. Patient and tumor characteristics were obtained and included patient’s age, date of tumor diagnosis, tumor staging according to AJCC 2017 [20], therapy approach, mortality (yes/no), recurrent disease (yes/no), and date of mortality/recurrent disease. Based on these parameters, we calculated the OS and DFS from the time of treatment (either surgery or the start of radio(chemo)therapy) to the last follow-up or an event (death or relapse, respectively). As the primary aim of the study was the assessment of the prognostic value of the SI, pretreatment values of absolute neutrophil and lymphocyte counts, serum albumin, as well as patient’s pretherapeutic height and weight were obtained using the hospital’s database system. Similarly, using the pretreatment computer tomography (CT) or magnetic resonance imaging (MRI) scans, we identified tumor localization and invasion with regard to OL. This assessment was performed by two coauthors (F.F.B. and L.K.-W.) and the concordance rate was analyzed. Exclusion criteria were prior treatment, secondary malignancy, incomplete follow-up data, missing laboratory values or ongoing immunosuppressive treatment.

All parameters contained in the SI were dichotomized into high (1) and low risk (0) and added to calculate the SI (0–6). Table 1 presents the SI parameters dichotomized into high and low risk. The cohort was stratified into low-risk (SI 0–2) and high-risk (SI 3–6) groups.

### Statistics

Based on the histograms of the individual parameters, we could assume a normal distribution of the data. Therefore, we use the mean ± standard deviation (SD) for presenting descriptive data. In order to compare OS and DFS between groups with low and high SI, we performed the log-rank test and graphically display these results with Kaplan–Meier survival curves. The survival times are presented as mean and 95% confidence interval (CI). As the cohort included a low number of patients and events, we did not perform multivariate analysis. The statistical significance level was set at *p* < 0.05. All *p* values had descriptive and hypothesis-generating characteristics. Therefore, no Bonferroni correction was performed. The statistical analysis included descriptive and survival statistics, and we plotted the results with the Statistical Package for the Social Sciences (SPSS, IBM Corp. Released 2016. IBM SPSS Statistics for Windows, Version 24.0. IBM Corp., Armonk, NY, USA).

## 3. Results

### 3.1. General Cohort Characteristics

A total of 51 patients with complete follow-up data, available pretreatment laboratory values, and data on pretherapeutic BMI (0–7 days prior to therapy) were identified. Patient demographics and tumor details are presented in Table 2. The majority of patients were men (*n* = 33, 64.7%), and the mean age of the whole cohort was 60.1 ± 12.9 years. A T3/T4 primary tumor was observed in 26 patients (51%), while the majority of patients had no regional lymph node metastases during the initial work-up (*n* = 40, 78.4%). Distant metastases were diagnosed in two patients (4.5%). The selected initial treatment for 27 patients (52.9%) was surgery, while 24 patients (47.1%) received primary radiotherapy or chemoradiotherapy.

The mean serum albumin was calculated for the whole cohort as 41.5 ± 5.0 g (gram)/dL (deciliter), and average absolute neutrophil and lymphocyte counts were 5.7 ± 1.7 Giga (G)/Liter (L) and 1.7 ± 0.7 G/L, respectively. Based on latter two, the mean NLR was calculated as 4.1 ± 2.5. The mean weight and height of all patients were 76.5 ± 17.6 kg and 1.7 ± 0.1 m, respectively; therefore, on average, the mean BMI of the entire cohort was 25.5 ± 5.6. Based on the pretherapeutic imaging (CT and/or MRI), OL was either infiltrated or the tumor was located superiorly/posteriorly in 28 patients (54.9%). Importantly, the concordance rate of the assessment of OL infiltration was 100% between the two coauthors (F.F.B. and L.K.-W.) who independently performed this analysis. Based on all individual parameters stratified according to Table 1, the SI was calculated by summing up the individual values. The low-SI group consisted of 24 patients (47.1%), and the high-SI group consisted of 27 patients (52.9%). Table 2 presents the general patient and tumor characteristics stratified for low and high pretreatment SI.

### 3.2. Survival

**Figure 1:**
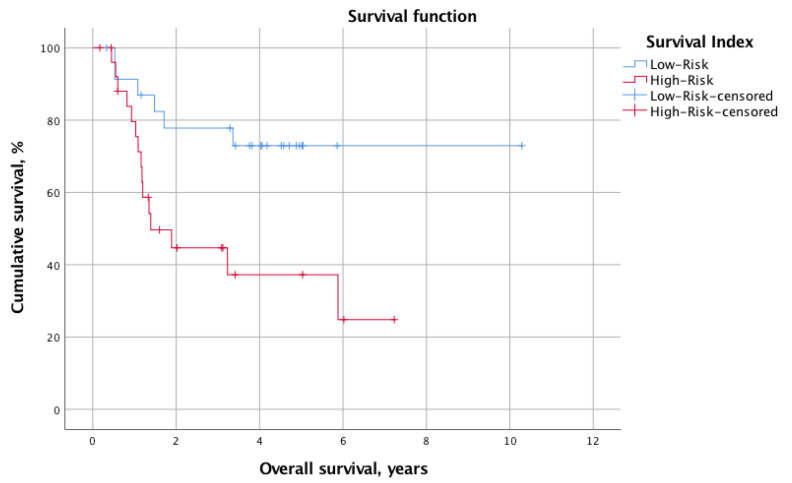
A Kaplan–Meier survival curve showing the OS for patients stratified into the low-SI (*n* = 24, SI 0–2) and high-SI (*n* = 27, SI 3–6) groups. The mean OS was shorter in the low-risk SI group (mean OS 7.9 years, 95% CI 6.3–9.6 years vs. 3.4 years, 95% CI 2.2–4.5 years). We tested it for statistical significance with the log-rank test, which revealed a significant difference in OS between groups (*p* = 0.013). OS, overall survival; SI, survival index; CI, confidence interval.

**Figure 2:**
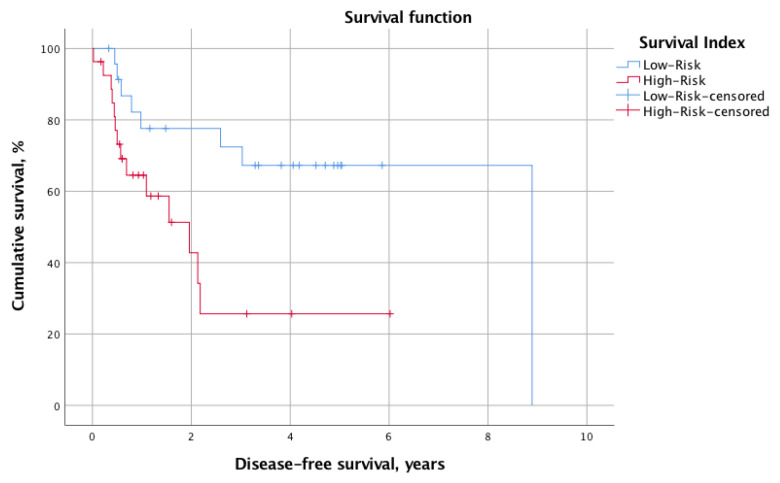
A Kaplan–Meier survival curve showing the DFS for patients stratified into the low-SI (*n* = 24, SI 0–2) and high-SI (*n* = 27, SI 3–6) groups. The mean DFS was shorter in the low-risk SI group (mean DFS 6.4 years, 95% CI 4.8–8.0 vs. 2.4 years, 95% CI 1.3–3.5). We tested it for statistical significance with the log-rank test, which revealed a significant difference in OS between groups (*p* = 0.013). DFS, disease-free survival; SI, survival index; CI, confidence interval.

**Figure 3:**
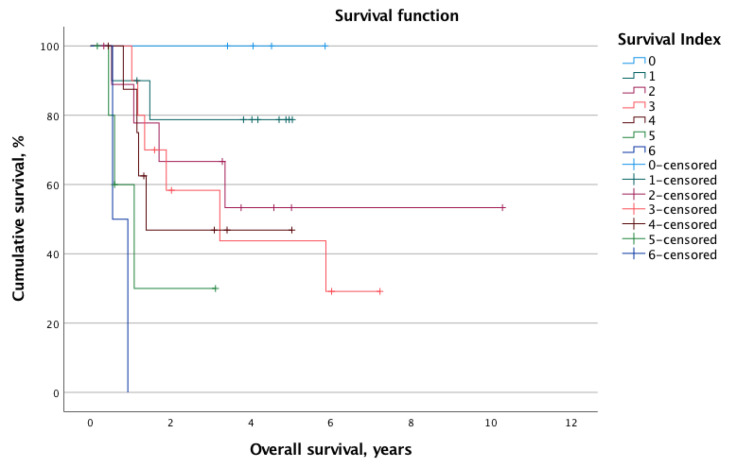
When stratified for the exact survival index score (0–6), the Kaplan–Meier survival curve shows a significant association between rising survival index and worse overall survival (*p* < 0.001).

**Figure 4:**
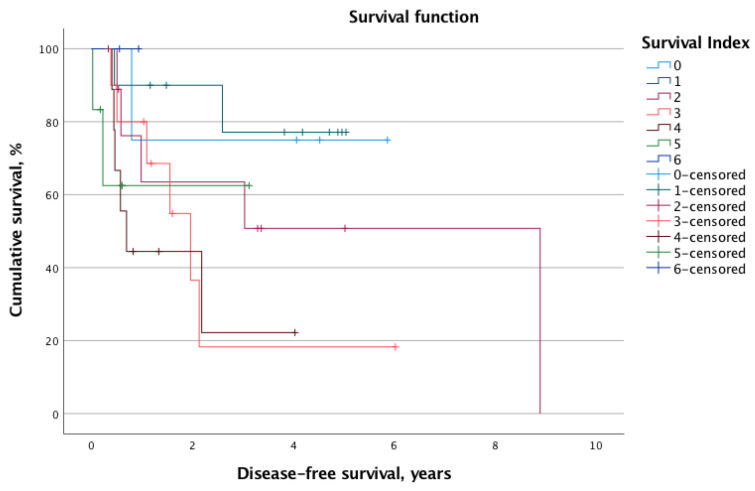
Similar to overall survival, the rising specific survival index score (0–6) was associated with worse disease-free survival. However, the correlation did not reach the level of statistical significance (*p* = 0.161).

The overall mortality and recurrence rates were 41.2% (*n* = 21) and 43.1% (*n* = 22), respectively. The mean calculated OS and DFS for the whole cohort were 2.8 ± 2.1 years and 2.1 ± 2.0 years, respectively. When stratified into low-risk (SI 0–2, *n* = 24, 47.1%) and high-risk (SI 3–6, *n* = 27, 52.9%) status according to the SI, the log-rank test revealed significantly different survival times. Indeed, the OS was significantly longer for the low-risk group (mean OS: 7.9 years, 95% CI 6.3–9.6 years vs. mean OS: 3.4 years, 95% CI 2.2–4.5 years; *p* = 0.013). Similarly, the DFS was prolonged in patients with low SI (mean DFS: 6.4 years, 95% CI 4.8–8.0 vs. mean DFS: 2.4 years, 95% CI 1.3–3.5; *p* = 0.012). Figure 1 and Figure 2 illustrate the Kaplan–Meier survival curves stratified for low- and high-risk SI with regard to OS and DFS, respectively. Moreover, Figure 3 and Figure 4 depict the OS and DFS stratified for precise SI score (0–6).

## 4. Discussion

Cancers of the paranasal sinuses are rare, and the anatomy in this region is very delicate. In particular, margin assessment is much more difficult due to endoscopic approaches than in other head and neck cancers. Thus, important information for further therapeutic decisions are often not available. Early stratification of patients into low- and high-risk groups with the help of novel tools to evaluate each patient’s prognosis can facilitate patient counseling and decision making with regards to therapeutic options and the frequency of clinical follow-up.

In our study, we proposed a novel prognostic marker that combines the prognostic relevance of already well-established clinical parameters (T classification, *N* classification, and tumor invasion with regars to the OL), one inflammation marker (NLR), and two markers of malnutrition and cachexia (BMI and albumin). Indeed, we observed poorer outcome in SNSCC patients with a high-risk SI with regard to OS and DFS.

Local tumor size and the involvement of regional lymph nodes, as reflected by the T and *N* classifications, are included in the TNM classification, and their prognostic significance is well-established [20]. The T classification reflects the local tumor size and, according to the current edition of AJCC, it is important to differentiate between tumors arising from the maxillary cavity and tumors arising from the nasal and etmoid cavities [20]. In summary, the classification ranges from T1 to T4a, with the last indicating the infiltration of the orbit/eye and/or intracranial growth. The *N* classification marks the regional tumor infiltration, particularly the cervical lymph nodes. It is well-known that extranodal extension is a negative prognostic marker. Therefore, as soon as the capsule of the lymph node is ruptured and an extranodal invasion is observed in the pathohistological analysis, the pathologist has to classify the tumor as N3b. Notably, the range is from N1 to N3b [25]. In summary, incorporating the T and *N* classifications into our proposed prognostic marker seems crucial because they reflect the local and regional tumor spread and should therefore negatively correlate with the survival probability.

As aforementioned, the OL divides the maxillary sinus and passes from the medial border of the orbit to the mandibular arch [21]. Its tumor infiltration can easily be assessed by the initial pretreatment imaging (CT or MRI), further underlining the feasibility of utilization of its prognostic value. Tumor extension across the OL has been inconsistently used throughout the years for the staging of paranasal sinus malignancies. In particular, in the first edition of the AJCC, from 1977, the invasion of the tumor across the OL was used for the separation of different stages of maxillary sinus cancer (ethmoid sinus was not mentioned). This classification, albeit with slight alterations, was maintained until 1997. From then on, including in the current 8th edition of the AJCC, the OL has not been mentioned [21]. The current T classification of maxillary and ethmoid sinus malignancies does not recognize the OL [20]. Nevertheless, a recent study revealed the potential prognostic value of the invasion across the OL in patients with nasal and paranasal cavity malignancies [21]. Therefore, we incorporated the prognostic relevance of tumor infiltration across the OL into our newly developed SI.

Another component of our proposed prognostic score is the NLR. The ratio of neutrophils to lymphocytes obtained from the peripheral blood was shown to negatively predict survival outcome in different malignancies, including non-small-cell lung cancer [26], rectal cancer [27], and even adenoid-cystic carcinoma [28]. In addition to being an indicator of the immunological response, NLR should also reflect the degree of cancer-related inflammation [29]. As aforementioned, the research on the prognostic significance of NLR in cancer started when the role of leukocytes in cancer development was first investigated and leukocytes in the tumor environment were first discovered [18]. In addition to the noted studies in different malignancies, the prognostic capacity of NLR is already well-established for head and neck cancer. Interestingly, Homa-Mlak et al. even showed that NLR has prognostic value in terms of predicting oral mucositis in head and neck cancer patients undergoing radiotherapy [30].

Lastly, the two final constituents of the SI are albumin and BMI. These markers positively reflect the patient’s nutrition level from the peripheral, blood, and clinical perspectives [19,31]. Moreover, cancer-related systemic inflammation also results in loss of weight and cell mass, ultimately reflected by a lower BMI and decreased levels of serum albumin [30]. Importantly, hyposmia and anosmia are typical symptoms and/or postoperative complications in sinonasal tumors [32]. Indeed, it is well-established that anosmia alters eating habits and contributes to reduced appetite and, therefore, restricted calorie intake [33]. In total, low albumin levels and low BMI should therefore certainly negatively impact the survival in cancer patients. Specifically for albumin, the reduced intake of proteins ultimately results in lowered albumin synthesis. Notably, the prognostic relevance of BMI and albumin has already been shown for different malignancies [34,35,36,37,38]. The prognostic relevance of both of these has been shown for head and neck malignancies as well. First, Danan et al. investigated preoperative serum albumin in patients with surgically treated head and neck cancer [39]. Indeed, a significant association of low serum albumin level with a higher rate of wound infections and worse survival was observed. A similar observation was reported by Lim et al. for patients treated with systemic agents or radiotherapy [40]. In their prospective study on more than 300 patients, significant associations of low pretreatment serum albumin with worse OS, DFS, and cancer-specific survival (CSS) were observed. Interestingly, even cisplatin cytotoxicity was associated with low serum albumin. In particular, Ishizuka et al. conducted a study on 28 patients with head and neck cancer treated with cisplatin and observed a significant association of low serum albumin level with cisplatin-induced neutropenia [41].

In summary, the SI combines the prognostic values of well-established, clinical, inflammatory, and nutritional markers. These can be easily calculated and determined during the pretherapeutic work-up. Therefore, the determination of the SI requires no additional clinical investigations and can be easily performed because all SNSCC patients undergo a routine peripheral blood analysis, and have a clinical check-up (including weight and height measurement) and a radiological evaluation (CT or MRI) prior to the start of therapy. Furthermore, the assessment of OL infiltration seems very feasible and accurate, as the concordance rate between the two coauthors was 100%.

The practice of combining the prognostic capacities of several clinical or other prognosticators is new for SNSCC patients. As noted, several composite scores could be identified in the literature for cancer in general [22] as well as for specific malignant diseases. These include the marker reported by Repo et al. for patients with breast cancer [23]. Furthermore, Mahar et al. discussed this issue for patients with colorectal cancer [24]. Based on these studies, our study group made efforts to combine different, already well-established prognostic markers for SNSCC. Indeed, by combining clinical, nutritional, and inflammation markers, we proposed the SI and were able to show its promising prognostic value with regard to OS and DFS. The feasibility of this marker should be further underlined, as all calculations and measurements needed for SI can be taken from routinely performed clinical and radiological work-up prior to the start of any cancer therapy.

Although our study provides novel evidence of the prognostic role of our combined score SI, we acknowledge that our results are constrained by some limitations. First, due to the retrospective nature of the study, potential selection bias could not be excluded. Regarding the statistical analyses, due to the limited number of events in the survival analysis, no multivariate investigations were possible. Therefore, the effects of potential confounders (therapy regimen, T and *N* classification, age, sex, stage, etc.) could not be analyzed. Nevertheless, we included a uniform cohort of squamous cell carcinoma of nasal and paranasal cavities, and the number of patients was relatively high given the low prevalence of this tumor entity. Therefore, our study should serve as a basis for future research of the prognostic relevance of the SI in SNSCC.

## 5. Conclusions

The present study provides evidence of the prognostic value of the newly proposed SI in patients with SNSCC. As the expected survival outcome in SNSCC patients remains poor, identifying high-risk patients is crucial in order to accordingly adapt therapy and follow-up regimes. As the SI can be easily calculated from blood samples, CT or MRI scans, and clinical examinations performed prior to therapy start, it might represent a clinically useful and easily obtainable prognostic marker. Further studies investigating the prognostic relevance of SI are warranted.

## Figures and Tables

**Table 1:** Stratification of individual SI components into low- and high-risk groups. For every parameter, a patient can be assigned either 0 (low-risk) or 1 (high-risk), and the SI is the sum of all the individual parameters binary value. SI, survival index; OL, Ohngren’s line; NLR, neutrophil-to-lymphocyte ratio; BMI, body-mass index; g/dL, gram/deciliter.

SI Component	Low Risk	High Risk
T classification	T1 and T2	T3 and T4
*N* classification	N0	N+
OL	Tumor inferior to OL	Tumor superior and/or across the OL
NLR	≤3	>3
BMI	≥25	<25
Albumin	≥35 g/dL	<35 g/dL

**Table 2:** Detailed tumor and patient characteristics stratified according to the pretreatment SI. SI; survival index, SD; standard deviation, M; male, F; female.

	Low SI (*n* = 24)	High SI (*n* = 27)	Cohort (*n* = 51)
Age			
Mean, years	58.7	61.4	60.1
SD, years	12.9	14.6	12.9
Sex, *n*/%			
M	18/75.0	15/55.6	33/64.7
F	6/25.0	12/44.4	18/35.3
T classification, *n*/%			
T1	9/37.5	0/0.0	9/17.6
T2	7/29.2	2/7.4	9/17.6
T3	1/4.2	5/18.5	6/11.8
T4	7/29.2	19/70.4	26/51.0
Tx	0/0.0	1/3.7	1/2.0
*N* classification, *n*/%			
N0	22/91.7	18/66.7	40/78.4
N1	0/0.0	0/0.0	0/0.0
N2	1/4.2	7/25.9	8/15.7
N3	0/0.0	1/3.7	1/2.0
Nx	1/4.2	1/3.7	2/3.9
M classification, *n*/%			
M0	23/95.8	26/96.3	49/96.1
M1	1/4.2	1/3.7	2/3.9
Primary therapy, *n*/%			
Surgery	18/75.0	9/33.3	27/52.9
Radio ± chemotherapy	6/25.0	18/66.7	24/47.1

## Data Availability

The data presented in this study are available on request from the corresponding author.
